# The Velvet Protein UvVEA Regulates Conidiation and Chlamydospore Formation in *Ustilaginoidea virens*

**DOI:** 10.3390/jof8050479

**Published:** 2022-05-04

**Authors:** Mina Yu, Junjie Yu, Huijuan Cao, Xiayan Pan, Tianqiao Song, Zhongqiang Qi, Yan Du, Shiwen Huang, Yongfeng Liu

**Affiliations:** 1 State Key Laboratory of Rice Biology, China National Rice Research Institute, Hangzhou 311400, China; 20130030@jaas.ac.cn (M.Y.); huangshiwen@caas.cn (S.H.); 2 Institute of Plant Protection, Jiangsu Academy of Agricultural Sciences, Nanjing 210014, China; jjyu@jaas.ac.cn (J.Y.); caohuijuan@jaas.ac.cn (H.C.); panxy@jaas.ac.cn (X.P.); tianqiao.song@njau.ac.cn (T.S.); 20130019@jaas.ac.cn (Z.Q.); 20130011@jaas.ac.cn (Y.D.)

**Keywords:** rice false smut, *Ustilaginoidea virens*, velvet, conidia, chlamydospores

## Abstract

Rice false smut, caused by *Ustilaginoidea virens*, is a serious disease of rice worldwide, severely reducing the quantity and quality of rice production. The conserved fungal velvet proteins are global regulators of diverse cellular processes. We identified and functionally characterized two velvet genes, *UvVEA* and *UvVELB*, in *U. virens*. The deletion of these genes affected the conidiation of *U. virens* but had no effect on the virulence of this pathogen. Interestingly, the Δ*UvVEA* mutants appeared in the form of smaller false smut balls with a reduced number of chlamydospores compared with the wide-type strains. In addition, the deletion of *UvVEA* affected the expression of some transmembrane transport genes during chlamydospore formation and rice false smut balls development. Furthermore, the Δ*UvVEA* mutants were shown to be defective in the utilization of glucose. These findings proved the regulatory mechanism underlying the formation of rice false smut balls and chlamydospores and provided a basis for the further exploration of the mechanism of these processes.

## 1. Introduction

*Ustilaginoidea virens* (Cooke) Takah (Teleomorph: *Villosiclava virens*) is the causal agent of rice false smut (RFS) diseases, which causes quantitative and qualitative losses in rice production. In recent years, RFS has emerged as one of the most devastating diseases of rice worldwide, including locations such as India, the Middle East, and North America [1]. In China, with the lack of high-level resistance in the existing rice germplasm, the annual average incidence of RFS is 3.06 million ha, resulting in a yield loss of 158.6 million kilograms per year [1]. In addition to causing severe yield losses, *U. virens* predominantly produces two types of mycotoxins, ustiloxins and ustilaginoidins, which are harmful to the nervous system of animals, and exert inhibitory effects on the growth of plants [2,3]. During infection in rice spikelets, *U. virens* hijacks rice nutrients, and transforms individual grains into so-called smut balls only at the booting stage. The mature rice false balls contain numerous yellow or dark green chlamydospores. This is the most important symptom caused by *U. virens* in rice. Chlamydospores, acting as one of the surviving propagules in natura, play an important role in the epidemiology of seasonal RFS disease [4].

During its infection on rice spikelets, *U. virens* hyphae preferentially attack stamen filaments. Subsequently, the hypha spreads and reaches anthers, lodicules, stigmas, and ovaries, and gradually forms false smut balls. No specialized infection structures have been found in this infection process [1]. Practices such as breeding for disease resistance and the use of protective fungicide spray are being routinely used to control RFS. However, the lack of major resistance genes in rice creates great difficulty in disease control. Therefore, molecular determinants of virulence in this pathogen remain to be fully elucidated and characterized in order to develop resistance to false smut disease in rice.

Many developmental and virulence-related genes have been identified and characterized from *U. virens* [1]. Among them, transcription factors play a crucial role in development and virulence, regulating the expression of downstream genes directly or indirectly. The transcription factors UvPRO1, UvCom1, and UvCCHC5 play critical roles in hyphal growth, conidiation, stress response, and virulence [5,6,7]. A homeobox transcription factor, UvHOX2, is needed for chlamydospore formation and virulence in *U. virens* [8]. UvMSN2, a C2H2-type zinc finger transcription factor, also plays an important role in the conidiation, stress responses, vegetative growth, and virulence of *U. virens* [9], as well as in the Zn(2)-Cys(6) class fungal-specific transcription factor UvZnFTF1 [10]. The cutinase G-box binding transcription factor UvCGBP1, which is unique to ascomycetes, regulates the transcription of the MAPK pathway kinase gene *UvSlt2*, involved in virulence and hyphae growth in *U. virens* [11]. Although many transcription factors were identified and characterized, the biology and virulence mechanisms of most transcription factors still remain poorly understood in *U. virens*.

The velvet family proteins, a class of fungal transcription factors, comprise a conserved velvet domain with proline residues in the middle of the motif [12]. In filamentous fungi, members of the velvet protein family are global regulators of a variety of cellular processes, such as fungal development, resting structure formation, and the production of secondary metabolites. The first velvet family member described was velvet A (VeA), as a positive regulator of sexual reproduction and a negative regulator of asexual development in the ascomycete *Aspergillus nidulans* [13]. Further studies showed that the homologs, veA in *Aspergillus flavus*, and BcVEA in *Botrytis cinerea*, were negative regulators of conidiation [14,15]. In contrast, the corresponding *veA* genes were positive regulators of conidiation, as is the case in *Aspergillus parasiticus*, *Fusarium fujikuroi*, and *Fusarium verticillioides*, and hyphal fragmentation, and as is the case in *Acremonium chrysogenum* [16,17,18,19]. Furthermore, VeA proteins have been found to play important roles in virulence in many pathogenic fungi, including *Magnaporthe oryzae* [20], *Fusarium graminearum* [21], *B. cinerea* [15], *Fusarium oxysporum* [22], *Valsa mali* [23], and *Verticillium wilt* [12]. Vel1 is necessary for initial plant root colonization in *Verticillium dahliae* [12]. Taken together, this evidence suggests important regulatory roles for VeA in diverse aspects of fungal biology. VelB in *A*. *nidulans* or other ascomycetes has similar functions to those of VeA [12,15,18]. Taken together, these pieces of evidence suggest important regulatory roles for VeA and VelB in diverse aspects of fungal biology and virulence. However, the roles of VeA and VelB proteins in *U. virens* are still unknown.

In this study, we hypothesized that UvVEA and UvVELB, in the role of VeA and VelB homologues, would regulate fungal development, conidiation, and virulence, and be a potential target to combat the fungus outside or inside rice. To address this hypothesis, the functions of these two genes were analyzed by a target gene deletion strategy. We characterized the roles of UvVEA and UvVELB in growth, conidiation, and virulence in *U. virens*.

## 2. Materials and Methods

### 2.1. Fungal Strains and Growth Conditions

*U. virens* wide-type strain Jt209 is a single-conidium strain, isolated from infected rice spikelets collected in Jiangsu, China in 2018. The strains were confirmed as *U. virens* by morphological and molecular methods. *U. virens* strains were routinely maintained in potato sucrose agar (PSA: 200 g/L potato, 20 g/L sucrose, and 15 g/L agar) or potato sucrose (PS: 200 g/L potato and 20 g/L sucrose) medium at 28℃ in the dark. After 10 d of growth on PSA plates, 5 mm diameter mycelial plugs of the strains were collected for phenotypic analysis.

### 2.2. Bioinformatic Methods

The *U. virens* protein database was downloaded from the NCBI. Typical velvet domains of predicted proteins were verified through CD search (Conserved Domain Database, http://www.ncbi.nlm.nih.gov/Structure/cdd/wrpsb.cgi (accessed on 4 July 2019)). Sequence analysis of proteins was performed with the InterPro website. Nuclear localization signals (NLS) and PEST sequence prediction were performed with cNLSmapper and epestfind, respectively (http://emboss.bioinformatics.nl/cgi-bin/emboss/epestfind (accessed on 11 September 2021)), with default settings [12]. The information regarding the conversed domain, intron and exon structures, and the positional information of the CDS sequences, was downloaded from the NCBI and loaded by the Gene Structure Display Server (GSDS) 2.0 (http://gsds.cbi.pku.edu.cn (accessed on 20 September 2021)) [24]. The phylogenetic tree of different velvet proteins was constructed by the neighbor-joining method using MEGA 7.0 (Philadelphia, PA, USA) (accessed on 18 October 2021).

### 2.3. Target Gene Deletion and Complementation in U. virens

To explore the biological role of *UvVEA* and *UvVELB* in *U. virens*, approximately 1000 bp of the downstream and upstream flanking sequences of the genes and the hygromycin resistance cassette were ligated into the pMD19 T-simple vector. Then, we generated gene deletion mutants by replacing the full-length fragment of the genes with a hygromycin resistance cassette using the CRISPR/Cas9 system (Figures S3 and S4) [25]. For the complementation assays, a fragment containing an approximately 1.5 kb native promoter region and the full-length gene sequence were ligated into the pKO1 vector [26]. *Agrobacterium tumefaciens* strain AGL-1 harboring the pKO1-*UvVEA* or pKO1-*UvVELB* constructs were transformed into conidia of the Δ*UvVEA* or Δ*UvVELB* mutants, respectively, by *Agrobacterium tumefaciens*-mediated transformation [27]. G418-resistant transformants were generated and confirmed by both PCR and RT-qPCR analysis.

### 2.4. Vegetative Growth and Conidiation

For vegetative growth assays, 5 mm mycelial plugs were transferred and grown on fresh YT agar medium (0.1% yeast extract, 0.1% tryptone, and 1% glucose) at 28 °C. After 12 d of incubation, the colony diameter was measured. Conidiation capacity was assayed in liquid YT cultures initiated with five mycelial plugs. After shaking at 160 rpm for 7 d at 28 °C in the dark, conidial production was measured using a hemocytometer. To observe conidium generation structures, 20 μL (10^6^ conidia/mL) of conidia was cultured on water agar plates for 10 d [8]. Conidium generation structures were observed under the microscope. All the experiments were performed three times with three replicates. 

For the sugar utilization assays, mycelial plugs were inoculated on the plates with Czapek–Dox agar medium containing different sugar sources (30 g/L), including glucose, sucrose, lactose, maltose, raffinose, stachyose, trehalose, and soluble starch [6,7]. Followed by a 12-day incubation period in the dark at 28 °C, the radial growth of vegetative mycelia was measured, and the inhibition of the radial growth was calculated. The inhibitions were represented by the relative growth inhibition of the fungal colony with the formula (*D*c − *D*t)/*D*c × 100%, where *D*c and *D*t denote the diameter of the control and sugar colonies, respectively. All the experiments were performed three times with three replicates.

### 2.5. Virulence and Infection Assays

For virulence assay, strains were conducted on a susceptible rice cultivar LYP9 [27]. The virulence strains were tested according to the method described previously [28]. The mycelial plugs were placed into PS liquid cultures and shaken at 160 rpm for 7 d. Then, the cultures were homogenized in a blender. These mycelial and conidial suspensions (1 × 10^6^ conidia/mL, 2 mL) were inoculated into swollen sheaths of flag leaves on the main stems of rice at the late booting stage (approximately one week before heading) by a syringe [27]. Inoculated rice panicles were sampled at 1, 2, 3, 4, 5, 7, 10, and 14 dpi (days post inoculation) for reverse transcription–quantitative polymerase chain reaction (RT-qPCR). The number of false smut balls of each panicle was counted at 21 d after inoculation. Each strain was inoculated onto 15 panicles each time.

The morphology of false smut balls was examined after the inoculation of rice panicles with strains at 10, 14, 21, 28, or 35 dpi. The sizes of the smut balls were determined as the width and length from 20 conidia of each strain. For the dry weight of smut balls, 20 balls were collected randomly from an inoculated rice spikelet, and dried in an 80 °C oven until a stable weight was reached. For the number of chlamydospores, 4 g of smut balls was collected and soaked in 2 mL of sterile water. After shaking at 160 rpm for 2 h, the number of chlamydospores in the water were counted using a hemocytometer. Each treatment was repeated three times.

### 2.6. Nucleic Acid Manipulations and qRT-PCR

Fungal genomic DNA was isolated using the CTAB method from vegetative hyphae. Total RNA was isolated from 3-day-old vegetative hyphae or inoculated rice panicles using an RNA Extraction kit (Biotech, China) according to the manufacturer’s instructions. cDNA synthesis was performed using a PrimeScript RT reagent Kit with gDNA Eraser (TaKaRa). RT-qPCR was conducted using SYBR *Premix Ex Taq*^TM^ II Kit (TaKaRa) in a QuantStudio3 (Thermo Fisher) to detect the gene expression levels. The *β-tubulin* sequence or the rice Glyceraldehyde-3-Phosphate Dehydrogenase gene (*OsGAPDH*), respectively, was chosen as the endogenous reference for *U. virens* or rice [29]. The relative mRNA amounts were calculated by the −2^ΔΔCt^ method as described previously [28]. The PCR primers used in this study are listed in Table S1. 

### 2.7. Determination of the Interaction by Yeast Two-Hybrid Assay

The yeast two-hybrid system was utilized to identify the protein–protein interactions (Clontech, San Francisco, CA, USA). The coding sequences of the full-length *UvVEA* and *UvVELB* were amplified from the Jt209 cDNA with specific primers (Table S1) and inserted into the yeast GAL4 binding domain vector pGBKT7 and the GAL4 activation domain vector pGADT7, respectively, using the ClonExpress II One Step Cloning Kit (Vazyme, China). The yeast two-hybrid plasmids were co-transformed into the *Saccharomyces cerevisiae* reporter strain Y2HGold by the LiAc/SS Carrier DNA/PEG transformation procedure. The pair of plasmids pGADT7 and pGBKT7–53 served as a positive control. The pair of plasmids pGADT7 and pGBKT7-Lam was used as a negative control. The transformants were grown on SD/-Leu/- Trp, and SD/-Ade/-His/-Leu/-Trp medium for 3-5 days at 30 °C. The experiments were repeated three times independently to confirm the results.

### 2.8. Statistic Analysis

All the data from the experiments of three replicates were subjected to a one-way Analysis of Variance (ANOVA) and carried out with the SAS system (Cary, NC, USA) to find the difference in means between the deletion mutant and control strains. Data are shown as the mean ± standard deviation (SD).

## 3. Results

### 3.1. Identification and Characterization of UvVEA and UvVELB in U. virens

**Figure 1:**
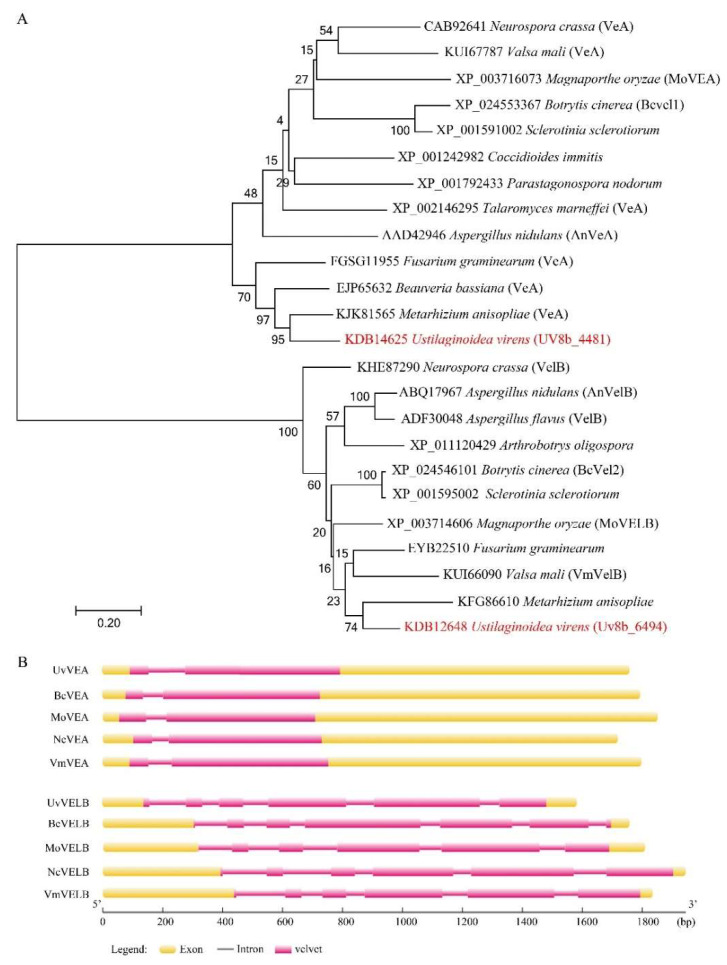
Velvet proteins are conserved among different fungi. (**A**) The phylogenetic trees for *UvVEA* and *UvVELB* genes and their orthologs in other species. Phylogenetic trees were created by neighbor-joining analysis with 1000 bootstrap replicates. The accession number in the GenBank database and the fungal species are labeled on the right. (**B**) Schematic diagram of the conserved motifs, and exon and intron structures of these genes. Yellow boxes represent exons, spaces among the boxes represent introns. Pink boxes represent a conserved velvet domain in the protein.

VeA and VelB homologs were initially identified through a homology search of the *U. virens* protein database by BLASTP search with the known velvet gene homologs *AnVEA* (XP_658656.1) and *AnVELB* (XP_657967.1) in *A. nidulans* as the query. Two homologs, named *UvVEA* (UV8b_01329) and *UvVELB* (UV8b_04876), were identified in this study (Figure 1A). 

To confirm the open reading frame of these two genes, the cDNA of Jt209 was amplified by PCR with gene-specific primers. *UvVEA* includes two exons and one intron, resulting in a transcript of 1629 base pairs (bp) with a deduced 542-amino-acid (aa) protein, which is similar to *AnVeA* (573 aa), sharing 52.74% identity. UvVEA carried a N-terminal velvet domain and a predicted nuclear localization signal (NLS) between amino acids 477 and 487, as in VdVEL1 and AnVeA at the C-terminal [12]. UvVEA contains a PEST domain between amino acids 406 and 420 but is different from the position described for AnVeA and VdVEL1 (Figure S1).

The original annotation of the *UvVELB* gene resulted in a deduced 371 aa protein. The sequencing of the cDNA of Jt209 revised the cleavage sites of the second and the third exons. The corrected *UvVELB* (KDB12648) sequence includes six exons and five introns for a 1149 bp transcript with a deduced protein of 382 aa, which shares 49.40% identity with *AnVELB*. The velvet domain is located in the C-terminal of UvVELB, interrupted by an intrinsically disordered domain (IDD). A predicted nuclear localization signal (NLS) between amino acids 56 and 68 is located in UvVELB (Figure S2). These architectures are similar to Vel2/VelB orthologues from other filamentous fungi [12]. Sequence analysis showed that the architecture of these two velvet proteins is largely conserved between *U. virens* and other ascomycetes (Figure 1B).

### 3.2. Roles of UvVEA and UvVELB Genes in Mycelial Growth and Conidiation in U. virens

To characterize the functional roles of velvet genes in the development and virulence of *U. virens*, the *UvVEA* or *UvVELB* deletion strains were constructed by replacing their open reading frames with a hygromycin resistance cassette. All the deletion mutants were confirmed by PCR and RT-qPCR analysis (Figures S3 and S4). 

**Figure 2:**
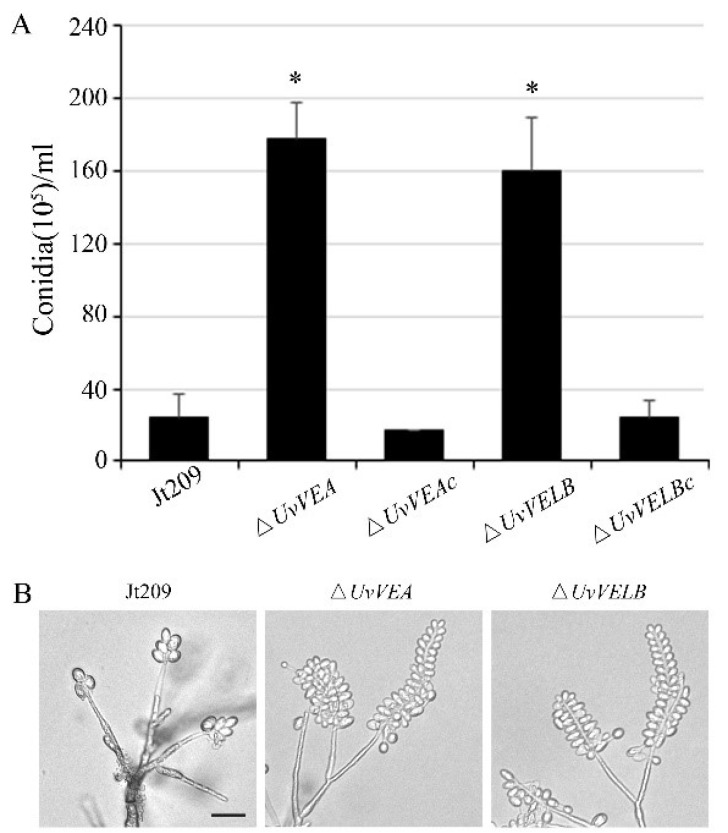
Comparisons of conidiation among Jt209, Δ*UvVEA*, and Δ*UvVELB* mutants. (**A**) Conidia were quantified after incubation of the wild-type strain (Jt209), Δ*UvVEA* mutants, Δ*UvVELB* mutants, and complemented strains in 50 mL YT liquid medium for 7 days in a shaker. Line bars denote standard deviation of three experiments. Asterisks (*) represent significant differences relative to the number of conidia in Jt209 (one-way ANOVA, α= 0.05). (**B**) Sporulation structures of Jt209, Δ*UvVEA* mutants, and Δ*UvVELB* mutants.

To investigate whether these two genes are associated with mycelial growth and conidiation, we performed a detailed analysis of the phenotypes of the wide-type strain Jt209, Δ*UvVEA* and Δ*UvVELB* mutants. As presented in Figure S5, the deletion of *UvVEA* and *UvVELB* did not significantly affect the growth rate and colony morphology. The production of conidia and conidial germination were then examined. After incubation in liquid YT medium with shaking for 7 days, the number of conidia produced by Δ*UvVEA* and Δ*UvVELB* mutants were 6.42- and 5.92-fold, higher than that of the Jt209, respectively (Figure 2A). To observe the intact conidial sporulating structures, we cultured the strains on MM media for 10 days in the dark. Microscopic observation of conidiation also revealed that all strains produced typical conidial sporulating structures, but both the Δ*UvVEA* and Δ*UvVELB* mutants produced more conidia on the sporulating structures (Figure 2B). However, the conidia produced by the Jt209 and the two deletion mutants showed a similar appearance, and appeared normal in terms of germination (Table S2). Phenotypic changes in the velvet mutants of *U. virens* were confirmed with complemented transformants. These results suggest that both velvet proteins are important for regulating conidial production in *U. virens*.

### 3.3. UvVEA and UvVELB Were Up-Regulated at Mycelial Expansion Stage during Infection

**Figure 3:**
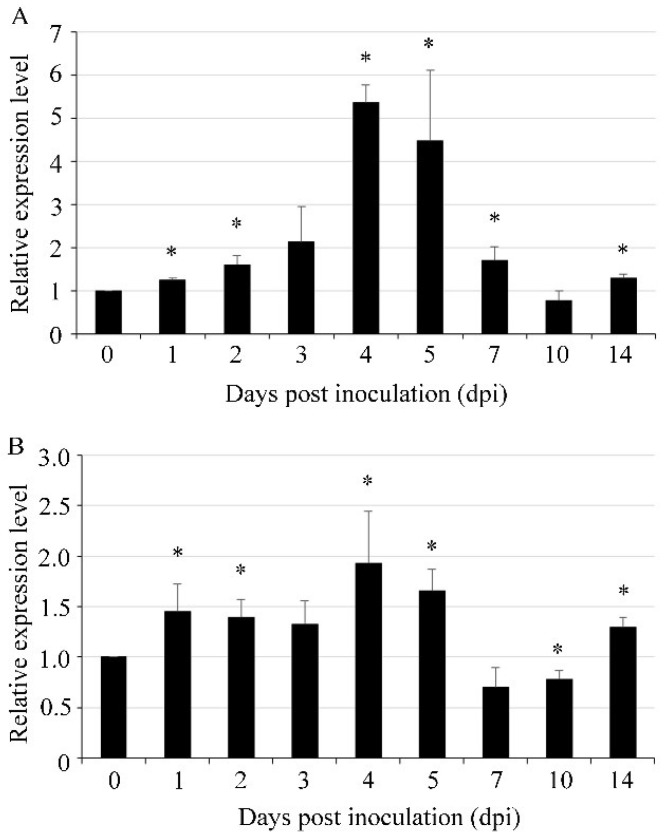
Expression of *UvVEA* and *UvVELB* genes in *U. virens*. Expression profiles of *UvVEA* (**A**) and *UvVELB* (**B**) genes in hyphae (0 dpi) and at different stages of infection in rice spikelets (1–14 d), as determined by RT-qPCR. Line bars indicate standard deviation of three experiments. Asterisks (*) represent the expression levels during infection significant differences relative to the control (hyphae) (one-way ANOVA, α = 0.05).

The gene expression profiles of *UvVEA* and *UvVELB* of *U. virens* during pathogenesis were determined by RT-qPCR at different expansion stages. In comparison with the vegetative mycelial stage in axenic culture, the expression level of *UvVEA* started to increase at 1 dpi. The expression peak was at 4 dpi, when *U. virens* invaded the floral organs of rice spikelets [30] with more than a 5-fold increase (Figure 3A). The expression pattern of the *UvVELB* gene during the infection processes was similar to that of *UvVEA* with a peak at 4 dpi (Figure 3B). These results suggest that *UvVEA* and *UvVELB* were likely involved in the infection of *U. virens*.

### 3.4. Roles of UvVEA and UvVELB in Virulence

**Figure 4:**
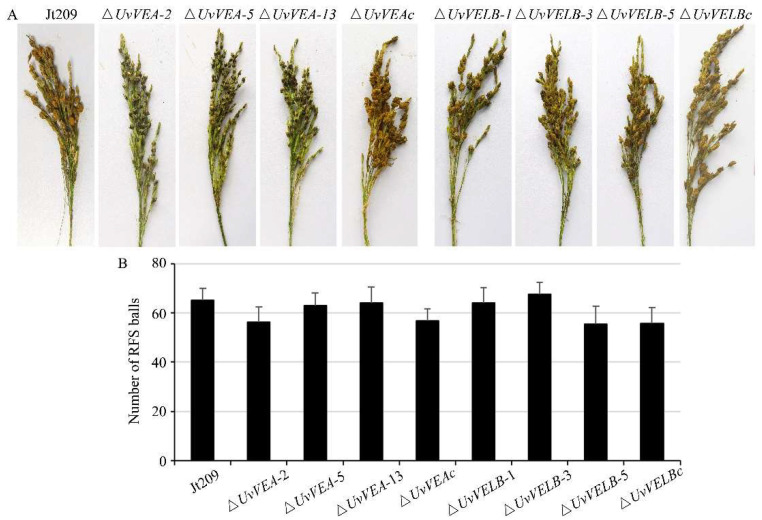
Impact of *UvVEA* and *UvVELB* gene deletion on virulence of *U. virens*. (**A**) Virulence assays of Jt209, the Δ*UvVEA* and Δ*UvVELB* mutants, and complementation strains on rice spikelets at 21 dpi. (**B**) Average number of smut balls per panicle. Each strain was inoculated onto 15 panicles each time. Line bars indicate the standard deviation of three experiments.

To investigate whether *UvVEA* and *UvVELB* were associated with virulence in *U. virens*, we performed virulence assays to analyze the capacity of the mutants to infect rice spikes by inoculating conidial and mycelium suspensions. At 10 dpi, consistent with the wild-type strain, dense hyphae of the Δ*UvVELB* mutants were in tight contact with the surfaces of filaments, anthers, stigmas, and ovaries in colonized rice (Figure S6). Three weeks after inoculation, the number and the morphology of rice false smut balls of the Δ*UvVELB* mutants were similar to those of Jt209 (Figure 4). Furthermore, the morphology and germination of chlamydospores of the Δ*UvVELB* mutants were also similar to those of Jt209. These results indicate that *UvVELB* has no effect on the infection, virulence, or chlamydospore development of *U. virens*.

**Figure 5:**
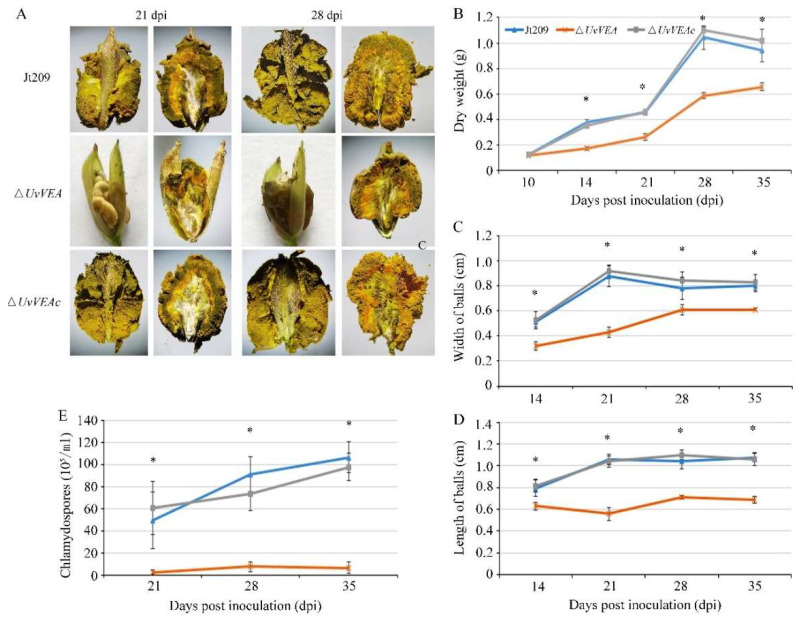
UvVEA is a key transcription factor governing smut ball and chlamydospore formation in rice. (**A**) Process of infection in inoculated rice spikelets. During infection, the dry weight (**B**), the size of rice false balls (**C**,**D**), and the number of chlamydospores (**E**) were decreased compared with Jt209. The sizes of rice false smut balls were determined as width and length from 50 conidia of each strain. Line bars in each column denote standard deviation of three experiments. Asterisks (*) represent the characteristic of rice false balls of the mutants significant differences relative to that of the wide-type strain Jt209 (one-way ANOVA, α = 0.05).

Interestingly, although the Δ*UvVEA* mutants showed a similar number of rice false smut balls to Jt209 and complemented transformants, the size of the false smut balls was significantly reduced compared with that of the wild-type strains (Figure 5A). Therefore, we observed the infection process of Jt209, and the Δ*UvVEA* mutants was observed in more detail. At 10 dpi, hyphae of both strains were observed on the surfaces of filaments in colonized rice (Figures S6 and S7). This indicated that the Δ*UvVEA* mutants successfully infected the rice spikelets, and *UvVEA* has no effect on the infection of *U. virens*. At 14 dpi, rice grains infected by the Δ*UvVEA* mutants contained small ball-like colonies between two glumes, whereas infection by Jt209 formed smut balls (Figure S7). At 21 and 28 dpi, rice grains infected by the Δ*UvVEA* mutants formed smut balls, but the sizes of these balls were smaller than those of Jt209 (Figure 5C,D). The sizes of the balls remained stable after 28 dpi. Consistently, the smut balls of the Δ*UvVEA* mutants had a lower dry weight (Figure 5B). 

There are a large number of yellow or dark green chlamydospores on the surface of mature false head smut balls, which is important in the epidemiology of RFS disease across seasons. We further measured the number of chlamydospores on the surface of the smut balls. The smut balls of the Δ*UvVEA* mutants contained fewer chlamydospores than those of Jt209 (Figure 5E). A longitudinal section of mature false smut balls showed that, at 21 dpi, the yellow hypha and chlamydospore of Δ*UvVEA* mutants were only present at the top of the balls, whereas for Jt209, they appeared across the entire surface of the ball (Figure 5A). At 28 dpi, the yellow hypha and chlamydospore layers of the Δ*UvVEA* mutants were significantly thinner than those of Jt209 (Figure S8). In addition, the morphology and germination of chlamydospores of the Δ*UvVEA* mutants were similar to those of Jt209. These results demonstrate that *UvVEA* was involved in the development of rice false smut balls and the formation of chlamydospores in *U. virens*.

### 3.5. Deletion of UvVEA Might Not Affect the Expression of Rice Grain-Filling-Related Genes

**Figure 6:**
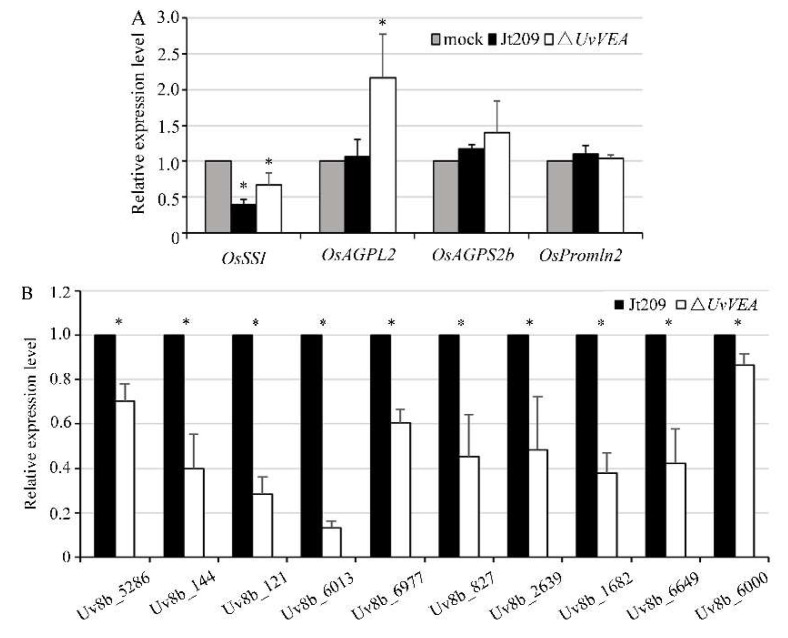
UvVEA governs transmembrane transporters of *U. virens*. (**A**) Expression of four rice grain-filling-related genes in rice at 14 dpi, as determined by qRT-PCR. The Ct value in the mock inoculation was set as a calibrator for each gene, which was carried out using PSB as inoculum. Asterisks represent significant differences relative to the mock inoculation (one-way ANOVA, α = 0.05). (**B**) Results of RT-qPCR to validate the expression of transmembrane transporter genes of *U. virens* in rice inoculated with Jt209 or the Δ*UvVEA* mutants in samples at 14 dpi. Asterisks (*) represent the expression level of genes in the Δ*UvVEA* mutants significant differences relative to that of the wide-type strain Jt209 (one-way ANOVA, α = 0.05). Line bars indicate standard deviation of three experiments.

As a biotrophic fungal pathogen, *U. virens* acquires nutrients via inducing the expression of genes associated with grain filling to mimic fertilization within the ovaries, providing large amounts of nutrients to the pathogen for false smut ball development [1,31]. In rice, grain-filling-related genes include seed storage protein genes and starch anabolism genes [29]. To determine whether *UvVEA* is involved in the regulation of genes related to grain filling and the formation of normal smut balls in rice, the expression of some grain-filling-related genes, including the starch metabolism genes *OsAGPL2, OsSSI,* F *OsAGPS2b*, and the seed storage protein gene *OsPromln2*, were detected between the WT and Δ*UvVEA* mutants [29]. The results show that the expression levels of *OsSSI* and *OsAGPL2* were upregulated (1.7–2.1-fold) in rice spikelets inoculated with the Δ*UvVEA* mutants, compared with the WT strain and the complementation strain. The expression levels of *OsAGPS2b* and *OsPromln2* were similar among the strains (Figure 6A). These results indicate that the deletion of *UvVEA* did not affect the expression of some rice grain-filling-related genes. 

### 3.6. UvVEA Plays Important Roles in Glucose Utilization, and Carbohydrate and Transmembrane Transport

We assumed that the defect of the Δ*UvVEA* mutants in forming normal smut balls was due to the impaired use of nutrients after *UvVEA* gene knockout, rather than the lack of a nutrient supply from rice. In order to confirm this hypothesis, we measured the expression level of genes related to the membrane transport and sugar transport of *U. virens* in rice false balls at 14 dpi, when the smut balls formed from the Δ*UvVEA* mutants were significantly smaller than those of Jt209. Various genes were significantly downregulated in the Δ*UvVEA* mutants during infection, including *Uv8b_6013* (MFS multidrug transporter), *Uv8b_6977* (Sugar transporter family protein), *Uv8b_144* (Carboxylic acid transport protein), *Uv8b_6649* (MFS transporter), *Uv8b_827* (Oligo peptide transporter), *Uv8b_5286* (MFS transporter), *Uv8b_121* (MFS transporter), *Uv8b_2639* (MFS transporter), *Uv8b_1682* (MFS transporter), and *Uv8b_6000* (MFS transporter) (Figure 6B) [6,7]. These results suggest that the Δ*UvVEA* mutants had defects in rice nutrient transportation and utilization, which prevented them from carrying out normal smut ball formation.

**Figure 7:**
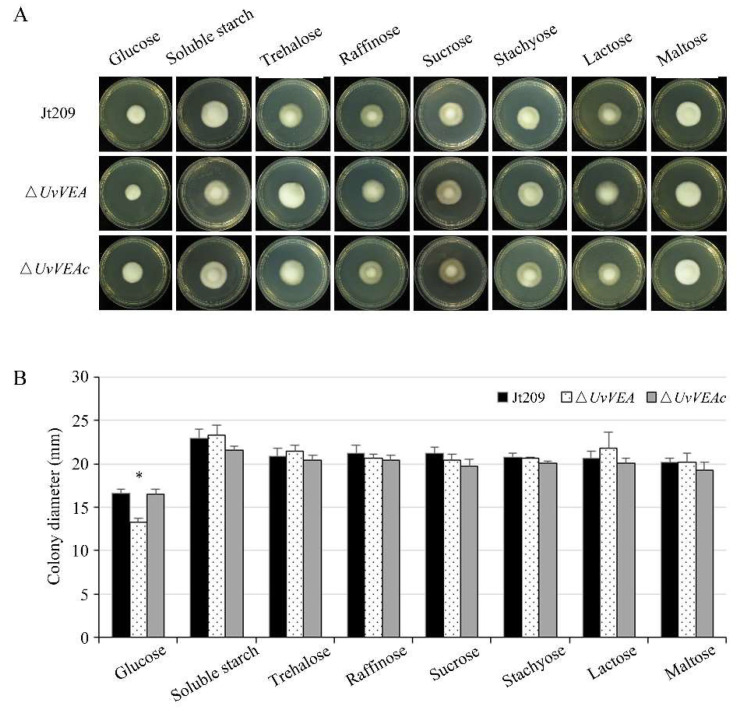
The Δ*UvVEA* mutants have defects in glucose utilization. (**A**) Colony morphology of Δ*UvVEA* mutants at 12 d of culture on plates containing different saccharides. (**B**) Colony diameter of Δ*UvVEA* mutants at 12 d of culture on plates containing different saccharides. Asterisks (*) represent the colony diameters of the Δ*UvVEA* mutants significant differences relative to that of the wide-type strain Jt209 (one-way ANOVA, α = 0.05). Line bars indicate standard deviation of three experiments.

Furthermore, we compared the carbohydrate utilization preferences of Δ*UvVEA* with those of Jt209 and the complementation strain Δ*UvVEAc*. We measured the radial growth rates of these strains on Czapek–Dox Agar medium containing different saccharides. Compared with Jt209 and Δ*UvVEAc*, the growth rates of the Δ*UvVEA* mutants were significantly reduced only on glucose media, suggesting that the mutants had defects in glucose utilization (Figure 7). These results indicate that *UvVEA* was involved in the utilization of glucose by *U. virens*.

### 3.7. UvVEA Plays Important Roles in BrlA-AbaA-WetA Regulatory Pathway

**Figure 8:**
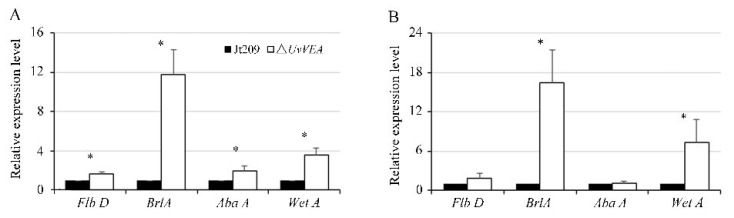
Expression of genes during conidiation or chlamydospore formation in *U. virens*. The relative expression level of *Flb D*, *Brl A*, *Aba A*, and *Wet A* in wide-type strain Jt209 and the Δ*UvVEA* mutants at initial stage of conidiation (**A**) or chlamydospores formation. (**B**) Line bars indicate standard deviation of three experiments. Asterisks (*) represent the expression level of genes in the Δ*UvVEA* mutants significant differences relative to that of the wide-type strain Jt209 (one-way ANOVA, α = 0.05).

In *Aspergillus* conidia, Myb-like DNA-binding protein FlbD delivers signals to activate the conidiogenesis regulatory cascade BrlA-AbaA-WetA [32]. In *Metarhizium robertsii*, AbaA interacts with the promoter regions of *VeA* and *WetA*, contributing to the separation of blastospores in submerged culture [33]. Therefore, we tested the expression levels of the homologs of *FlbD* (KDB18803), *BrlA* (KDB11753), *AbaA* (KDB11305), and *WetA* (KDB15008) in *U. virens* at the initial stage of chlamydospore and conidia formation [8]. The results show that these genes were expressed at a higher level in Δ*UvVEA* mutants than in Jt209 during both stages (Figure 8). This suggested that the BrlA-AbaA-WetA regulatory pathway may be involved in the generation of chlamydospores and conidia.

### 3.8. Interaction of UvVEA with UvVELB 

**Figure 9:**
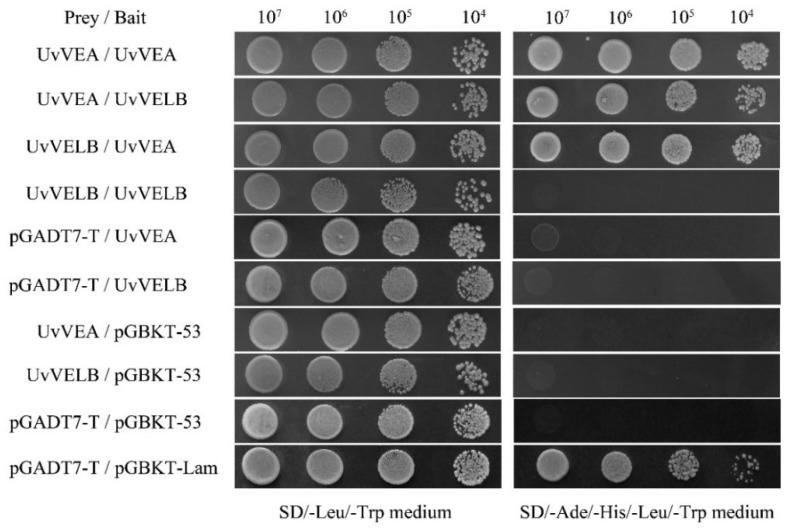
Yeast two-hybrid assay for the analysis of interaction between UvVEA and UvVELB proteins from *U. virens*. Yeast two-hybrid assay was used to examine the interaction between UvVEA and UvVELB proteins. Yeast strain Y2HGold expresses the indicated proteins fused to the DNA-binding domain (pGBKT7-53) or activation domain (pGADT7-T) of Gal4. Yeast cells of transformants were spotted in serial dilutions (10^7^–10^4^ cells/mL) containing prey and bait vectors on SD/-Leu/-Trp or on SD/-Ade/-His/-Leu/-Trp medium and incubated at 30 °C for 3 days. pGADT7-T/pGBKT-53, positive interaction control; pGADT7-T/pGBKT-Lam, negative interaction control. Three repeats were performed, and similar results were obtained.

In *A. nidulans* and *B. cinerea*, VelB physically interacts with VeA [14,15]. In this study, the yeast two-hybrid (Y2H) experiment was executed to test whether this interaction also occurs in *U. virens*. As shown in Figure 9, UvVEA was able to interact with UvVELB in the Y2H assay. Furthermore, UvVEA can interact with itself.

## 4. Discussion

Rice false smut, caused by *U. virens*, is one of the most devastating rice diseases worldwide. However, our understanding of the mechanisms of virulence of *U. virens* is limited because of its infection organ specificity and relatively difficult genetic transformation. In the current study, we identified and functionally characterized UvVEA and UvVELB proteins in *U. virens*, finding that both proteins were involved in conidiation. We further found that *UvVEA* gene deletion caused defects in the chlamydospore and false smut ball formation of *U. virens*.

A previous study showed that velvet proteins regulated filamentous growth and conidiation in many fungal species. In *A. nidulans*, the *VeA* mutant showed suppressed hyphal growth accompanied by vigorous conidiation [13,34]. The disruption of *FvVE1* led to reduced hyphal growth and increased conidiation in *F. verticillioides* [19]. The same effects have also been found in *FgVELB* in *F. graminearum* [21]. By contrast, the *FfVEL2* deletion mutants exhibited reduced hyphal growth and conidiation in *F. fujikuroi* [18]. The growth rate and conidiation of Δ*MoVEA* and Δ*MoVELB* in *M. oryzae*, and Δ*AovelB* in *Arthrobotrys oligospora*, were also significantly retarded [20,35]. In this study, the results suggest that UvVEA and UvVELB were dispensable for hyphal growth, but negatively regulated conidiation, a finding similar to results obtained from *B. cinerea* [15] and *V. mali* [23]. These results suggest that the velvet-protein-mediated mediated mechanisms controlling hyphal growth and conidiation in fungi are varied.

Furthermore, in many fungal species, the hydrophobic property of the cell surface is a significant feature of aerial hyphae. The Δ*Fvve1* and Δ*FgVelB* mutants showed a decreased hydrophobicity on the hyphal surface [19,21], suggesting their essential role in maintaining the hydrophobicity of the hyphal surface. To confirm the deduction on UvVEA and UvVELB, 20 μL aliquots of bromophenol blue were dropped on the colony surface of *U. virens* [21]. Bromophenol blue maintained spherical droplets on the surface of the mycelium of wild-type and complemented strains for at least 2 h, revealing the strong hydrophobicity of *U. virens* hyphae. However, similar to the wild-type and complemented strains, bromophenol blue also maintained spherical droplets on the colony surface of Δ*UvVEA* and Δ*UvVELB* mutants (Figure S9). These results suggest that *UvVEA* and *UvVELB* were dispensable for the hydrophobicity of *U. virens*. Thus, there are pieces of evidence showing the species-specific regulatory mechanisms of VeA and VelB proteins in asexual development.

Many studies have confirmed that the central regulatory pathway associated with conidiation is highly conserved in fungi. Three transcription factors of this pathway, BrlA, AbaA, and WetA, coordinate conidiation-specific gene expression, and cause the marked increase in conidiation in *Fusarium graminearum* and *Penicillium digitatum* [36,37]. Mr-AbaA positively regulates conidiation by regulating the orthologous gene of velvet family Mr-veA in *Metarhizium robertsii* [33]. The VelB-VosA hetero-dimer plays a role of the negative feedback regulation of the *brlA* gene in the conidia [38]. Here, we found that *BrlA*, *AbaA*, and *WetA* were expressed at a high level in the deletion of the *UvVEA*. This suggested that *UvVEA* plays a role of the negative feedback regulation of the BrlA-AbaA-WetA regulatory pathway for conidiation in *U. virens*.

The roles of VeA-like and VelB-like proteins in virulence are different in fungi. In the apple pathogen *V. mali*, Δ*VeA* mutants displayed reduced virulence on their hosts [23]. In *M*. *oryzae,* Δ*MoVEA* mutants displayed defects in appressorium formation and disease development [20]. *V*. *dahlia* Vel1 shows similar roles to those of the *M*. *oryzae* proteins, but the Δ*VEL2* mutants have similar disease symptoms to those of the the wild type [39]. Nevertheless, in *F. graminearum, FgVELB* deletion mutants showed impaired virulence on flowering wheat heads [21]. The Δ*AoVelB* mutants exhibited non-pathogenicity on *A. oligospora* [35]. In the current study, we observed that the virulence of *UvVEA* and *UvVELB* deletion mutants was in accordance with that of the wide-type strains on rice. Interestingly, the sizes of the rice false smut balls formed by Δ*UvVEA* mutants were smaller than those of the wide-type strain. As a biotrophic fungal pathogen, *U. virens* may acquire a considerable amount of nutrients by hijacking the rice grain-filling process [1]. We speculated that the mutants might have a defect in establishing links with nutrient supply between the mutant fungus and rice. Gene expression assay results show that the Δ*UvVEA* mutants induced the expression of rice grain-filling genes such as Jt209, whereas some trans-membrane transport genes were significantly downregulated in the Δ*UvVEA* mutants during infection. These results indicate that *UvVEA* might be involved in the nutrient transfer process of *U. virens*. A similar situation was observed with the *UvCCHC5* and *UvCom1* genes, the mutants of which can infect the spikelets of rice, but fail to form smut balls [6,7]. This result suggests that further study of UvVEA might provide insights into the mechanism of smut ball formation of *U. virens*.

Chlamydospores, as a type of asexual spores, can be found in a number of fungi. chlamydospores formation is regulated by several genes. In the human pathogen *Candida albicans*, conserved bHLH regulatory factor EFG, homeobox transcription factor GRF10*,* universal transcription inhibitor NRG1, and HOG1 in the MAPK pathway were found to be involved in chlamydospore formation. The velvet gene *vel1* is critical for chlamydospore formation in the biocontrol fungus *Trichoderma virens* [40], as well as calcineurin in the plant pathogenic fungi *F. oxysporum* [41]. In *U. virens*, our study confirms that UvVEA is also involved in chlamydospore formation. Furthermore, UvHOX2 could coordinate the regulation of the downstream BrlA-AbaA-WetA cascade during chlamydospore formation in *U. virens* [8]. In the current study, the expression of *BrlA* and *WetA* was upregulated at the initial stage of chlamydospore formation in the Δ*UvVEA* mutants compared with the wild-type strain. However, the number of chlamydospores on the surface of the smut balls of the Δ*UvVEA* mutants was significant reduced. These suggested that regulatory pathways other than BrlA-AbaA-WetA pathway may also be involved in chlamydospore formation. 

Previous studies showed that starch and sucrose metabolism, phosphatase, kinase, and transcription factors were involved in chlamydospore formation in *Phanerochaete chrysosporium* [42]. Yuan et al. (2019) speculated that genes relating to glycogen, lipid, and mannan may be conducive to energy storage and cell wall construction in chlamydospores in *Trichoderma harzianum* [43]. When the concentration of glucose exceeds 3%, it induced the transition from yeast-like cells to chlamydospores in *Aureobasidium pullulans* [44]. In this study, carbohydrate utilization assays showed that the Δ*UvVEA* mutants had the defect of using glucose. Thus, we speculated that the *UvVEA* gene is involved in glucose transport and affects the chlamydospore formation of *U. virens*. The roles of the proteins that interact with UvVEA in chlamydospore formation should be investigated in the future. Since chlamydospores are important surviving propagules for epidemic transmission of this pathogen, *UvVEA* could provide a novel molecular target for developing drugs against *U. virens* and offer a new theoretical basis for durable disease control strategies.

In summary, UvVEA and UvVELB are important for conidiation in *U. virens*. In addition, UvVEA also contributes to chlamydospore formation. Our findings improve our understanding of the regulatory mechanism underlying conidiation and chlamydospore formation in rice by *U. virens*.

## 5. Conclusions

In summary, this study is the first to uncover the roles of velvet proteins in regulating the development and virulence of *U. virens*. Our collective results demonstrate that *UvVEA* and *UvVELB* are crucial regulators of conidiation in *U. virens*. Moreover, Δ*UvVEA* mutants were shown to be defective in the utilization of glucose, and in the expression of some transmembrane transport genes during chlamydospore formation and rice false smut development. However, UvVELB appeared dispensable for the virulence of *U. virens*, unlike its homologs in other fungal species. Our results reveal the regulatory mechanism underlying the formation of rice false smut balls and provide a basis for the further exploration of the mechanism of these processes. Further investigation, including research into the protein–protein interactions of the *U. virens* velvet proteins, especially UvVEA, will provide insights into the development of chlamydospores and smut balls in this pathogen, and can be used to combat the growing threat of *U. virens* in rice.

## Data Availability

All experimental data in this study will be made available upon reasonable request from readers.

## Supplementary Materials

The following supporting information can be downloaded at: https://www.mdpi.com/article/10.3390/jof8050479/s1, Figure S1: Comparison of VeA-like proteins of *U. virens* and different fungi. Deduced VeA-like proteins of *Magnaporthe oryzae*, *Metarhizium anisopliae, Neurospora crassa*, *Aspergillus nidulans*, *Aspergillus flavus*, and *Botrytis cinerea*, similar to VeA of *U. virens* were aligned by DNASTAR using ClustalW multiple sequence alignment. The predicted velvet domain is depicted in light blue according to IPR037525. The predicted NLS is shown in purple and the PEST motif in green; Figure S2: Comparison of Velb-like proteins of *U. virens* and different fungi. Deduced Velb-like proteins of *Magnaporthe oryzae*, *Metarhizium anisopliae*, *Neurospora crassa*, *Aspergillus nidulans*, *Aspergillus flavus*, and *Botrytis cinerea*, similar to Velb of *U. virens* were aligned by DNASTAR using ClustalW multiple sequence alignment. The predicted velvet domain is depicted in light blue according to IPR037525. The predicted NLS is shown in purple; Figure S3: Deletion of the *UvVEA* gene in *U. virens*. (A) Illustration of targeted deletion of *UvVEA* utilizing CRISPR/Cas9 and homologous replacement. (B) PCR analysis of *UvVEA* deletion mutants Δ*UvVEA-2*, Δ*UvVEA-5*, and Δ*UvVEA-13*. The wild-type strain Jt209 was included as control. (C) Results of qRT-PCR to validate the expression of *UvVEA* gene in deletion mutants (Δ*UvVEA-2*, Δ*UvVEA-5*, and Δ*UvVEA-13*), the wild-type strain Jt209 and the complemented transformants; Figure S4: Deletion of the *UvVELB* gene in *U. virens*. (A) Illustration of targeted deletion of *UvVELB* utilizing CRISPR/Cas9 and homologous replacement. (B) PCR analysis of UvVELB deletion mutants Δ*UvVELB-1*, Δ*UvVELB-3*, and Δ*UvVELB-5*. The wild-type strain Jt209 was included as control. (C) Results of qRT-PCR to validate the expression of *UvVELB* gene in deletion mutants (Δ*UvVELB-1*, Δ*UvVELB-3*, and Δ*UvVELB-5*), the wild-type strain Jt209 and the complemented transformants; Figure S5: Hyphal growth of the UvVEA or UvVELB gene deletion mutants. (A) Colony morphology and mycelial growth of the strains were incubated on YT media for 12 days at 28 °C. (B) Colony diameter of the WT and the mutants on YT plates; Figure S6: Mycelial extension of the wide-type strain, the Δ*UvVEA* and Δ*UvVELB* mutants inside the spikelets at 10 dpi; Figure S7: Process of infection in inoculated rice spikelets. Infection progress of rice spikelets by the wide-type strain and the Δ*UvVEA* mutants at 10, 14 and 35 dpi, respectively.; Figure S8: The chlamydospore layer was thinner for false smut balls in the mutants than in Jt209. Figure S9: Role of velvet complex proteins in colony hydrophobicity. The indicated strains were grown in YT plates for 12 days at 28 °C in the dark. Twenty µL of water stained with bromophenol blue were dropped on the colony surface. Images were taken after 2 h, incubation at room temperature. During this time, the drop remains intact on hydrophobic colonies; Table S1: The primers used in this study; Table S2: Conidial sizes and germination of the Δ*UvVEA* and Δ*UvVELB* mutants.
