# Supplementary material for: The Velvet Protein UvVEA Regulates Conidiation and Chlamydospore Formation in Ustilaginoidea virens
Source: J Fungi (Basel). 2022 May 4;8(5):479. doi: 10.3390/jof8050479 (PMC9148152; doi:10.3390/jof8050479)
Supplement: Supplementary file 1 [file jof-08-00479-s001.zip › suppl figure lengend-N.pdf]

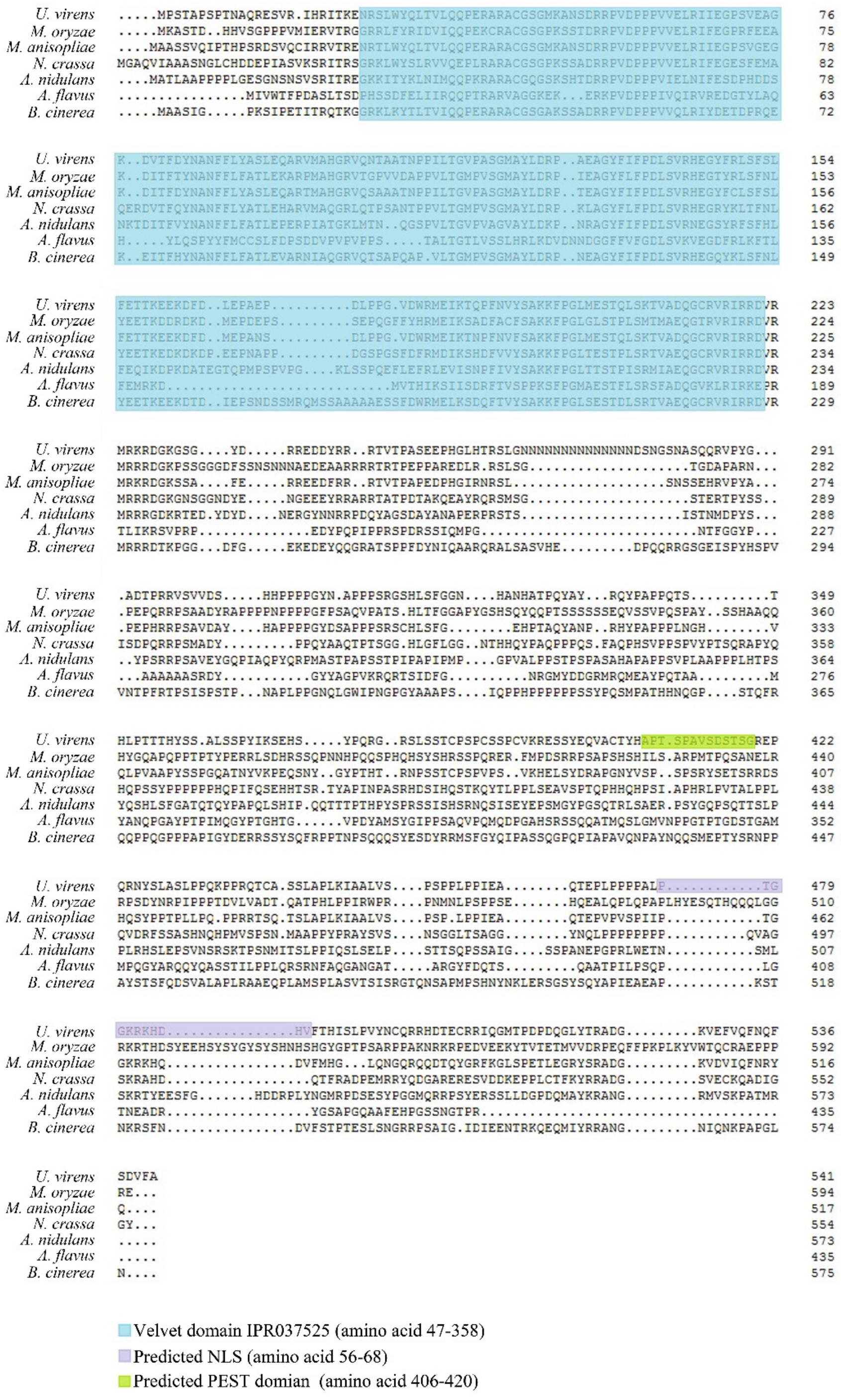

Figure S1. Comparison of VeA-like proteins of *U. virens* and different fungi.

Deduced VeA-like proteins of *Magnaporthe oryzae*, *Metarhizium anisopliae, Neurospora crassa*, *Aspergillus nidulans Aspergillus flavus*, and *Botrytis cinerea*, similar to VeA of *U. virens* were aligned by DNASTAR using ClustalW multiple sequence alignment. The velvet domain predicted by InterProScan for the UvVEA protein is depicted in light blue according to IPR037525. The predicted NLS is shown in purple and the PEST motif in green.

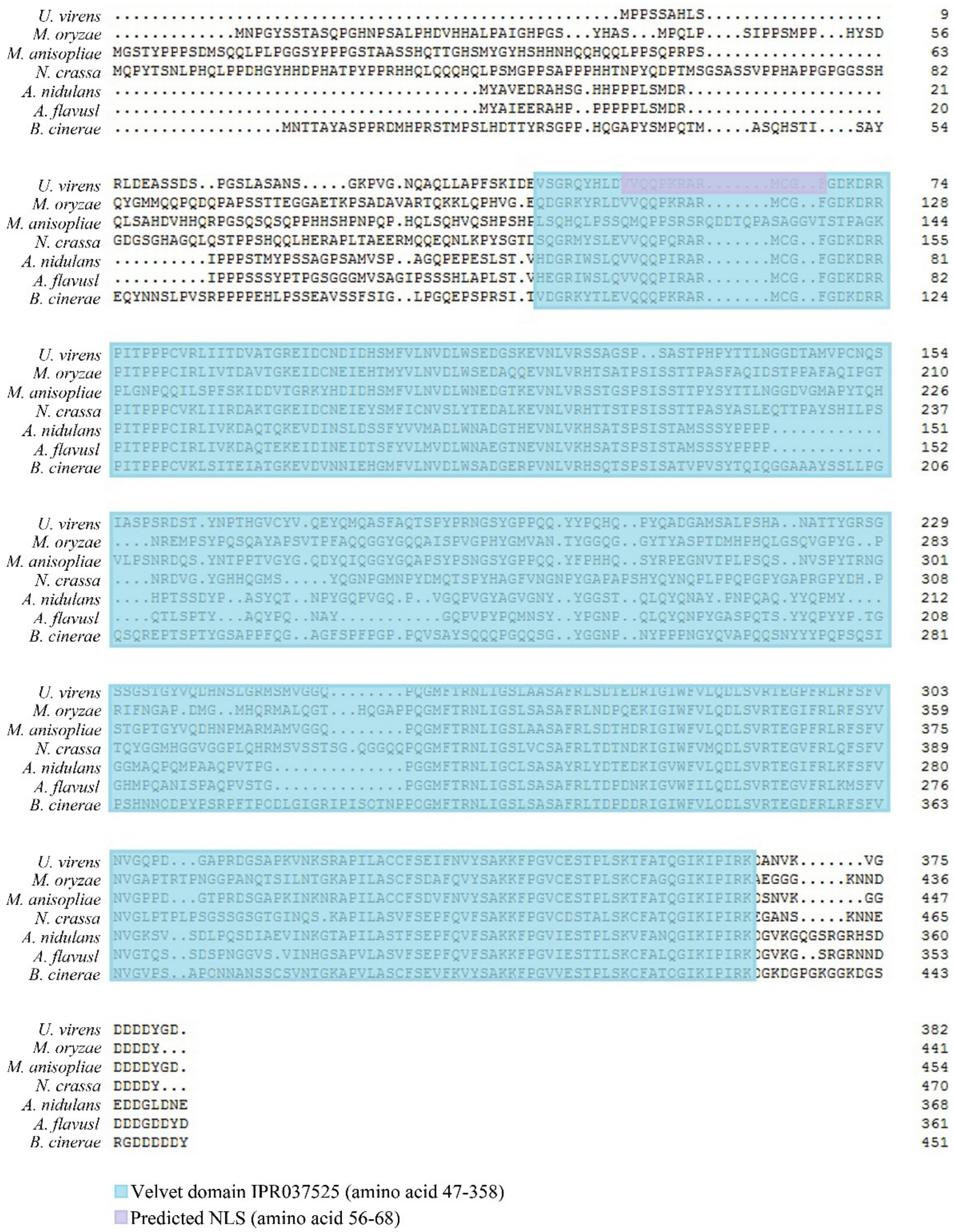

Figure S2. Comparison of Velb-like proteins of *U. virens* and different fungi.

Deduced VeA-like proteins of *Magnaporthe oryzae*, *Metarhizium anisopliae, Neurospora crassa*, *Aspergillus nidulans Aspergillus flavus*, and *Botrytis cinerea*, similar to Velb of *U. virens* were aligned by DNASTAR using ClustalW multiple sequence alignment. The velvet domain predicted by InterProScan for the UvVELB protein is depicted in light blue according to IPR037525. The predicted NLS is shown in purple.

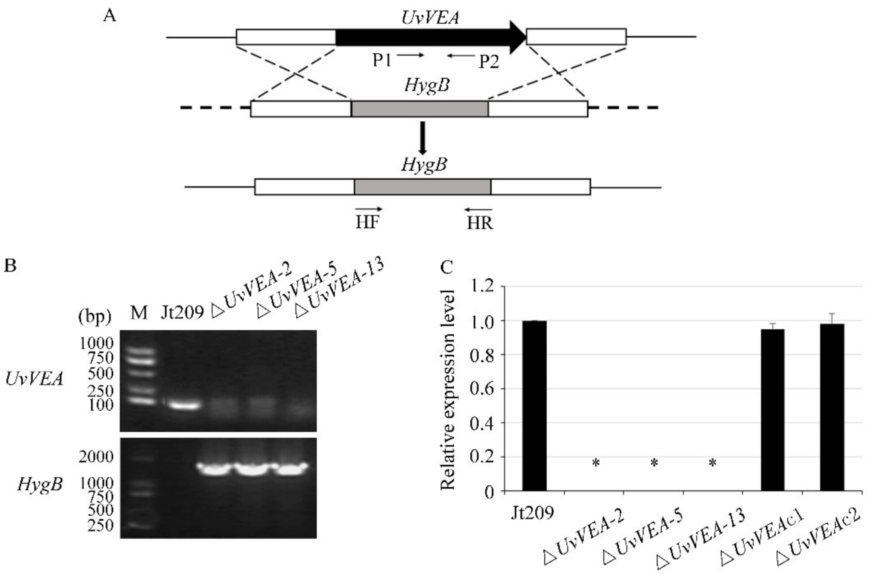

Figure S3. Deletion of the *UvVEA* gene in *U. virens*.

(A) Illustration of targeted deletion of *UvVEA* utilizing CRISPR/Cas9 and homologous replacement. (B) PCR analysis of *UvVEA* deletion mutants △*UvVEA-2*, △*UvVEA-5*, and △*UvVEA-13*. The wild-type strain Jt209 was included as control. (C) Results of qRT-PCR to validate the expression of *UvVEA* gene in deletion mutants (△*UvVEA-2*, △*UvVEA-5*, and △*UvVEA-13*), the wild-type strain Jt209 and the complemented transformants.

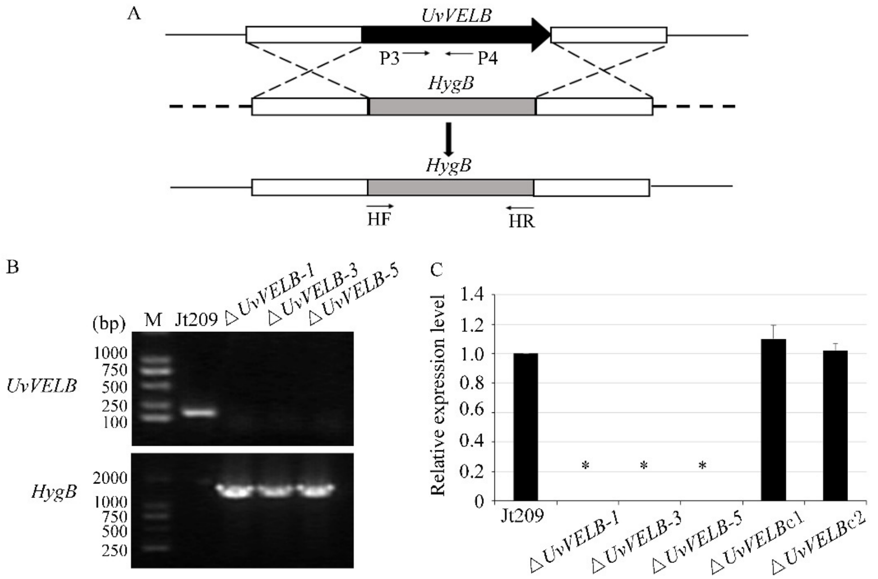

Figure S4. Deletion of the *UvVELB* gene in *U. virens*.

(A) Illustration of targeted deletion of *UvVELB* utilizing CRISPR/Cas9 and homologous replacement. (B) PCR analysis of *UvVELB* deletion mutants △*UvVELB-1*, △*UvVELB-3*, and △*UvVELB-5*. The wild-type strain Jt209 was included as control. (C) Results of qRT-PCR to validate the expression of *UvVELB* gene in deletion mutants (△*UvVELB-1*, △*UvVELB-3*, and △*UvVELB-5*), the wild-type strain Jt209 and the complemented transformants.

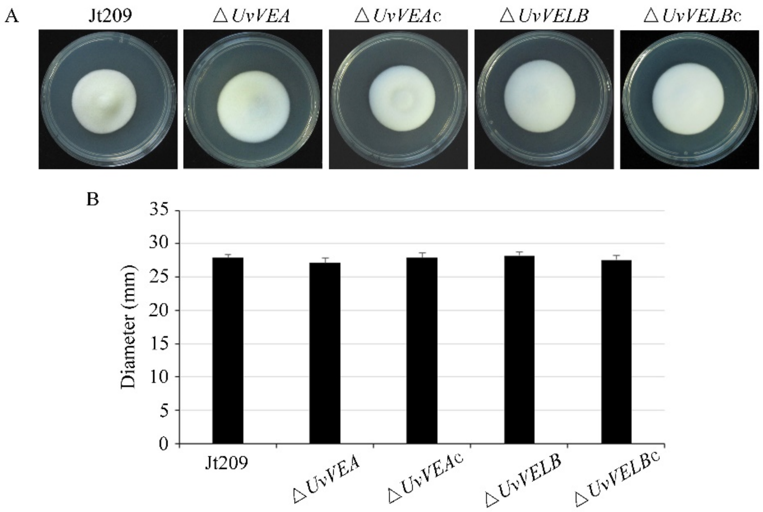

Figure S5. Hyphal growth of the *UvVEA* or *UvVELB* gene deletion mutants.

(A) Comparison of colony morphology and mycelial growth between the wild type (Jt209) and mutant lines. Colonies from the WT and mutant lines were incubated on YT media for 12 days at 28℃. (B) Colony diameter of the WT and mutants on YT plates.

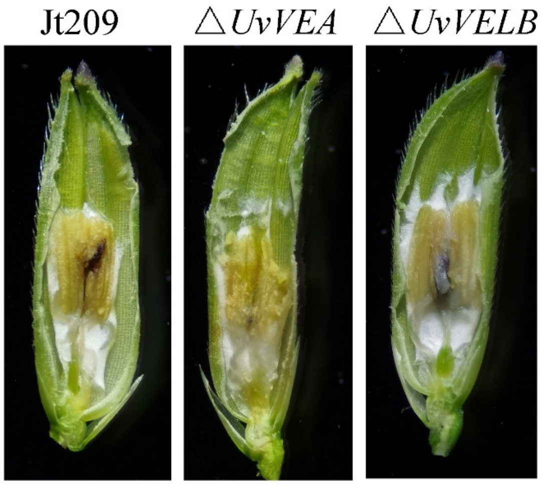

Figure S6. Mycelial extension of the wide-type strain, the Δ*UvVEA* and △*UvVELB* mutants inside the spikelets at 10 dpi.

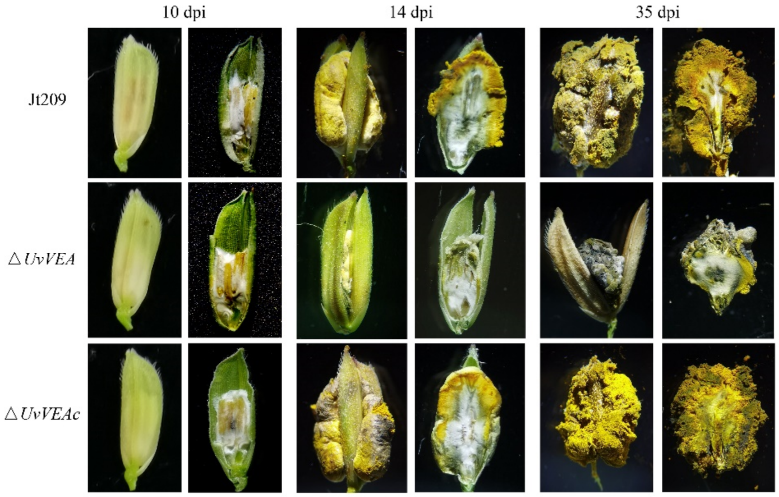

Figure S7. Process of infection in inoculated rice spikelets.

Infection progress of rice spikelets by the wide-type strain and *ΔUvVEA* at 10, 14 and 35 dpi, respectively.

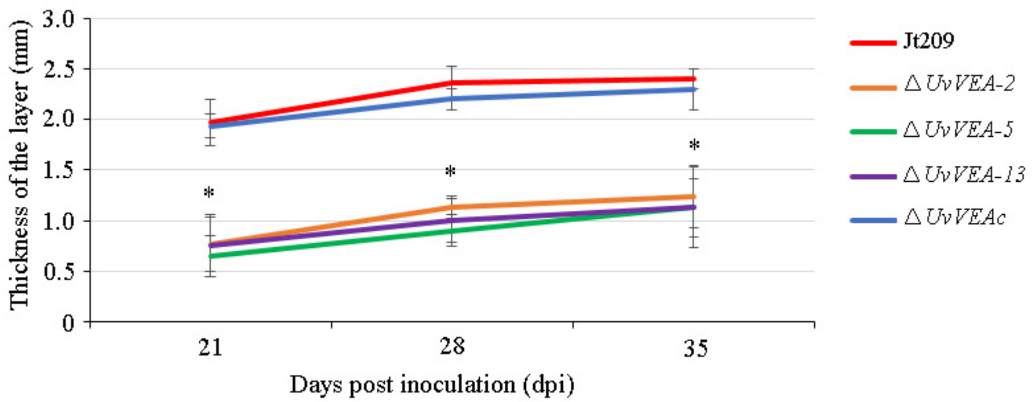

Figure S8. The chlamydospore layer was thinner for false smut balls in the mutants than in Jt209.

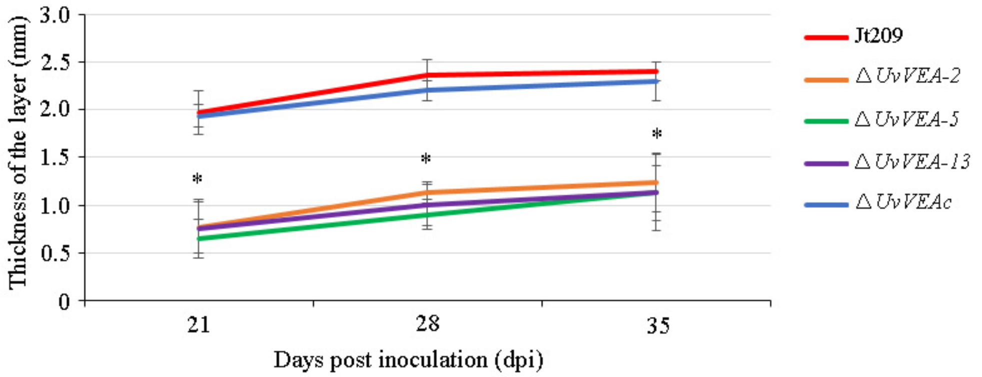

Figure S8. The thickness of the yellow hypha and chlamydospore layer of rice false smut balls.

The rice false smut balls were cut from the middle with a ascalpel. The thickness of the yellow hypha and chlamydospore layer of balls were measured with a ruler at its longitudinal section, at 21, 28 and 35 dpi, respectively. Line bars in each column denote standard deviation of three experiments. Asterisks represent significant differences relative to Jt209 (one-way ANOVA, α= 0.05)

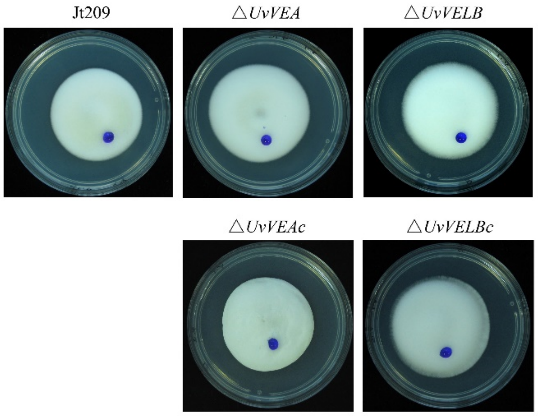

Figure S9. Role of velvet complex proteins in colony hydrophobicity.

The indicated strains were grown in YT plates for 12 days at 28ºC in the dark. Twenty µl of water stained with bromophenol blue were applied on the colony surface and images were taken after 2 hours, incubation at room temperature. During this time, the drop remains intact on hydrophobic colonies.
