# Supplementary material for: The Velvet Protein UvVEA Regulates Conidiation and Chlamydospore Formation in Ustilaginoidea virens
Source: J Fungi (Basel). 2022 May 4;8(5):479. doi: 10.3390/jof8050479 (PMC9148152; doi:10.3390/jof8050479)
Supplement: Supplementary file 1 [file jof-08-00479-s001.zip › Table S1 primers.pdf]

Table S1. The primers used in this study.

Primers	Sequence (5'-3')
qUvVEA F	CATGCAAACCATGCCACTCC
qUvVEA R	TACGGCGAGGATAGAGCAGA
UvVEA F	ATGCCGTCCACTGCTCCCAG
UvVEA R	TCATCCTGCAAAGACATCGGA
pKO-UvVEA F	TTCTGACCCGGGGATCCTCTCCTGCATCTCACTCGGT
pKO-UvVEA R	GGCCAGTGCCAAGCTTCACCGCCCCAACAATCCAC
UvVEA CR F	ACCTTCTCGAATAAGCTGAATGAC
UvVEA CR R	AAACGTCATTCAGCTTATTCGAGA
UvVEA KO1 F	GGATCTTCCAGAGATTCATCTCGTTCCTGTCAACC
UvVEA KO1 R	GCTCCTTCAATATCATCTTCTGTTCTGTCACTGTTCGCTGTG
UvVEA KO2 F	CGAGGGCAAAGGAATAGAGTACACGACAGTGACAGTGAGAGT
UvVEA KO2 R	CTGCCGTTCGACGATTCTTTGGAACCTGGATGGA
UvVEA AD F	GGAGGCCAGTGAATTCATGCCGTCCACTGCTCC
UvVEA AD R	CGAGCTCGATGGATCCTCATCCTGCAAAGACATC
UvVEA BK F	CATGGAGGCCGAATTCATGCCGTCCACTGCTCC
UvVEA BK R	GCAGGTCGACGGATCCTCATCCTGCAAAGACATC
qUvVELB F	CAACAAGAGCAGAGCTCCCA
qUvVELB R	GCCAACCTTGACGTTTGCAT
UvVELB F	ATGCCGCCGTCGTCGGCGCAC
UvVELB R	TCAATCCCCATAATCGTCGTC
pKO-UvVELB F	TTCTGACCCGGGGATCCAGTCGTACACGCGATAAGCC
pKO-UvVELB R	GGCCAGTGCCAAGCTTCGGTGCGAAATCAGAAATG
UvVELB CR F	ACCTCAAGAGCAGAGCTCCCATTC
UvVELB CR R	AAACGAATGGGAGCTCTGCTCTTG
UvVELB KO1 F	GGATCTTCCAGAGATTCGGTGCGAAATCAGAAATG
UvVELB KO1 R	GCTCCTTCAATATCATCTTCTGTTGTTGGAGGTGCGAGACAT
UvVELB KO2 F	CGAGGGCAAAGGAATAGAGTAGGCGATGACGACGATTAT
UvVELB KO2 R	CTGCCGTTCGACGATTTACAGTCCAAGCCTTTCACA
UvVELB AD F	GGAGGCCAGTGAATTCATGCCGCCGTCGTCGGC
UvVELB AD R	CGAGCTCGATGGATCCTCAATCCCCATAATCG
UvVELB BK F	CATGGAGGCCGAATTCATGCCGCCGTCGTCGGC
UvVELB BK R	GCAGGTCGACGGATCCTCAATCCCCATAATCG
