# Supplementary material for: The Velvet Protein UvVEA Regulates Conidiation and Chlamydospore Formation in Ustilaginoidea virens
Source: J Fungi (Basel). 2022 May 4;8(5):479. doi: 10.3390/jof8050479 (PMC9148152; doi:10.3390/jof8050479)
Supplement: Supplementary file 1 [file jof-08-00479-s001.zip › Table S2 Conidial sizes and germination rate of the íUvVEA and íUvVELB mutants.pdf]

Table S2. Conidial sizes and germination rate of the △*UvVEA* and △*UvVELB* mutants.

Strain	Conidial length (μm)[a]	Conidial wideth (μm)	Germination rate (%)[b]
Jt209	7.29 ± 0.60 A	3.89 ± 0.35 A	93.28 ± 1.33 A
△*UvVEA*	7.05 ± 0.23 A	3.92 ± 0.17 A	94.45 ± 2.37 A
△*UvVELB*	7.02 ± 0.47 A	3.86 ± 0.24 A	94.32 ± 1.84 A

a. The conidia sizes (length and wideth) were measured by microscope (50 conidia per strain).

b. Conidia were coated on YT medium. The conidia germination rates were calculated after 24 h incubation in the dark.

c. Data from at least three replicates were analyzed with the protected Fisher's least significant difference (LSD) test. Different letters mark statistically significant differences ($P \leq 0.01$).
